# Performance of the Large Language Model ChatGPT on the National Nurse Examinations in Japan: Evaluation Study

**DOI:** 10.2196/47305

**Published:** 2023-06-27

**Authors:** Kazuya Taira, Takahiro Itaya, Ayame Hanada

**Affiliations:** 1 Department of Human Health Sciences Graduate School of Medicine Kyoto University Kyoto Japan; 2 Department of Healthcare Epidemiology Graduate School of Medicine and Public Health Kyoto University Kyoto Japan; 3 Department of Preventive Medicine and Public Health School of Medicine Keio University Tokyo Japan

**Keywords:** ChatGPT, artificial intelligence, natural language processing, registered nurses, National Nurse Examination, Japan

## Abstract

**Background:**

ChatGPT, a large language model, has shown good performance on physician certification examinations and medical consultations. However, its performance has not been examined in languages other than English or on nursing examinations.

**Objective:**

We aimed to evaluate the performance of ChatGPT on the Japanese National Nurse Examinations.

**Methods:**

We evaluated the percentages of correct answers provided by ChatGPT (GPT-3.5) for all questions on the Japanese National Nurse Examinations from 2019 to 2023, excluding inappropriate questions and those containing images. Inappropriate questions were pointed out by a third-party organization and announced by the government to be excluded from scoring. Specifically, these include “questions with inappropriate question difficulty” and “questions with errors in the questions or choices.” These examinations consist of 240 questions each year, divided into basic knowledge questions that test the basic issues of particular importance to nurses and general questions that test a wide range of specialized knowledge. Furthermore, the questions had 2 types of formats: simple-choice and situation-setup questions. Simple-choice questions are primarily knowledge-based and multiple-choice, whereas situation-setup questions entail the candidate reading a patient’s and family situation’s description, and selecting the nurse's action or patient's response. Hence, the questions were standardized using 2 types of prompts before requesting answers from ChatGPT. Chi-square tests were conducted to compare the percentage of correct answers for each year's examination format and specialty area related to the question. In addition, a Cochran-Armitage trend test was performed with the percentage of correct answers from 2019 to 2023.

**Results:**

The 5-year average percentage of correct answers for ChatGPT was 75.1% (SD 3%) for basic knowledge questions and 64.5% (SD 5%) for general questions. The highest percentage of correct answers on the 2019 examination was 80% for basic knowledge questions and 71.2% for general questions. ChatGPT met the passing criteria for the 2019 Japanese National Nurse Examination and was close to passing the 2020-2023 examinations, with only a few more correct answers required to pass. ChatGPT had a lower percentage of correct answers in some areas, such as pharmacology, social welfare, related law and regulations, endocrinology/metabolism, and dermatology, and a higher percentage of correct answers in the areas of nutrition, pathology, hematology, ophthalmology, otolaryngology, dentistry and dental surgery, and nursing integration and practice.

**Conclusions:**

ChatGPT only passed the 2019 Japanese National Nursing Examination during the most recent 5 years. Although it did not pass the examinations from other years, it performed very close to the passing level, even in those containing questions related to psychology, communication, and nursing.

## Introduction

### What is ChatGPT?

ChatGPT is a large language model developed by OpenAI [1]. Based on the GPT architecture, it is capable of generating high-quality, human-like text in response to prompts. Pretrained on a large corpus of text data, it has been fine-tuned for specific natural language processing (NLP) tasks such as language generation and summarization. With several variants available, ChatGPT—the largest one containing over 175 billion parameters [2]—is one of the largest deep learning models in existence. Its potential applications include being used as a chatbot, language translation, text summarization, and content generation, making it a significant advancement in NLP.

### Application of ChatGPT to Medical Fields

Artificial intelligence (AI) applications have been used in the medical field, including medical chatbots, and applications that analyze and summarize electronic medical record systems, perform image diagnosis, analyze and organize the medical literature, and perform patient monitoring [3,4]. Release of the high-quality chatbot ChatGPT has also attracted attention in the field of medical education, as questions on the United States Medical Licensing Examination were reportedly answered with 60% accuracy, which is the threshold for passing the examination [5-7]. In addition, studies have evaluated the ChatGPT’s responses to questions on counseling for the treatment of infectious diseases [8] and prevention of cardiovascular diseases [9].

### Differences Between Physician and Nurse Specialties

Although physicians and nurses both play critical roles in the health care system, their specialties and responsibilities differ. Physicians focus on diagnosing and treating illnesses, whereas nurses focus on providing direct patient care and support. Nurses monitor patient health, administer medications, assist with activities of daily living, and provide emotional support to patients and their families. Nurses also communicate with other health care professionals to ensure that patients receive the appropriate care. Therefore, their training and responsibilities generally focus more on patient care and communication than on diagnosis and treatment.

### Evaluating the Performance of ChatGPT on the National Nurse Examinations in Japan

While passing the national examination does not guarantee the ability to practice in a clinical setting, a different scenario arises when considering the performance on registered nurse licensing examinations. These examinations feature questions that emphasize on patient emotions and communication, contrasting with those found in physician licensing examinations. Notably, if excellent performance can be demonstrated in these nursing examinations, it is likely to pave the way for a significant expansion of AI applications in the medical field. However, the performance of ChatGPT on nursing licensing examinations has not yet been evaluated.

We aimed to evaluate the performance of ChatGPT on national examinations for registered nurses in Japan.

## Methods

### Input Data Sets From the National Nurse Examinations in Japan

The data sets included questions and answers from the National Nurse Examinations in Japan from 2019 to 2023 (Multimedia Appendix 1). These examinations are conducted annually and include 240 multiple-choice questions, in which candidates are required to select 1 or, in some cases, multiple correct answers (ie, all that apply) from several options. These examinations were divided into morning and afternoon sessions, each comprising 120 questions. The questions covered 32 areas, including basic nursing skills, adult nursing, gerontological nursing, pediatric nursing, pathology, anatomy, and physiology. The Japanese National Nurse Examinations consist of 2 types of questions, basic knowledge and general questions, and all 240 questions must be answered. The basic knowledge questions are based on basic issues of particular importance to nurses, such as fundamental knowledge and basic nursing skills, while the general questions are based on the extensive knowledge of each nursing specialty, covering anatomy, physiology, and disease. As inappropriate questions are excluded from scoring, the criteria could change slightly; however, the passing criteria are 80% for basic knowledge questions and approximately 60% for general questions. In addition, the situation-setup questions included among the general questions were worth 2 points, whereas all other questions were worth 1 point. While the simple-choice questions are mainly multiple-choice knowledge questions, the situation-setup question requires the candidate to read a description of the situation of the patient and the patient's family, and then select the action to be taken by the nurse or the response to the patient.

### Data Exclusion

Each year, the Ministry of Health, Labor and Welfare (MHLW) of Japan, which certifies the qualification of registered nurses nationwide, reviews questions among conducted examinations, which cannot be answered with just 1 answer, or questions for which no correct answer exists, based on the MHLW’s own checks and comments from a third-party organization—the Japan Nursing School Association. Then, the MHLW deems these as “inappropriate questions” and removes them from the examinations. The inappropriate questions were excluded from this study. In addition, all questions were screened, and questions containing visual assets, such as clinical images, medical photography, and graphs, were removed because ChatGPT (GPT-3.5) is an interactive language AI that does not support image recognition.

### Prompt Engineering

Because prompt engineering significantly affects generative output, we standardized the input formats of the questions [10]. Question and answer prompts were created optimally based on the Prompt-Engineering-Guide published on GitHub [11] to achieve conservative performance rather than simply achieving the highest scores. As the National Nurse Examinations include 2 types of questions, 2 prompts were created (Textbox 1).

**Prompts for questions.**

Prompt 1: Simple-choice questions

Please answer the following questions briefly and by number.

Question: <Questionnaire contents>

1. <Option 1>

2. <Option 2>

3. <Option 3>

4. <Option 4>

Prompt 2: Situation-setup questions

Based on the following situation setup, please answer the questions briefly and by number.

Situation-setup: <Situation-setup contents>

Question: <Questionnaire contents>

1. <Option 1>

2. <Option 2>

3. <Option 3>

4. <Option 4>

### Data Analyses

Based on the scoring criteria of the official nursing examination, the percentage of correct answers provided by ChatGPT (GPT-3.5) was calculated separately for basic knowledge and general questions. We calculated the percentage of correct answers for each of the simple-choice questions (1 point, prompt 1) and the situation-setup questions (2 points, prompt 2) and conducted a chi-square test to compare the percentage of correct answers between the 2 prompts. Finally, the percentage of correct answers was calculated for each of the 32 subject areas, and areas with higher and lower percentages of correct answers compared with the overall mean and 1 SD were extracted. All statistical analyses were performed using R (version 3.6.2; R Foundation for Statistical Computing).

### Ethics Approval

This study did not require ethics approval because we only analyzed data from a published database.

## Results

### Input Data Statistics

Five years of the National Nurse Examination data showed that the largest number of inappropriate questions occurred in 2019, with 10 questions having been excluded from the scoring and 2 or 3 inappropriate questions in the other years. The number of questions with figures and tables ranged from 6 to 16. Thus, the number of questions analyzed in this study was 214 of 240 in the lowest year and 232 of 240 in the highest year (Table 1).

**Table 1:** Questions included and excluded in the analysis from 2019 to 2023.

Year	Included questions (mean 225.8, SD 6.2), n	Inappropriate questions (mean 4, SD 3)^a^, n	Questions with chart (mean 10.2, SD 3.4)^a^, n	Total, n
2019	214	10	16	240
2020	229	3	8	240
2021	228	2	10	240
2022	232	2	6	240
2023	226	3	11	240

^a^“Inappropriate questions” and “questions with chart” were excluded in the analysis.

### Evaluation Outcomes

**Figure 1:**
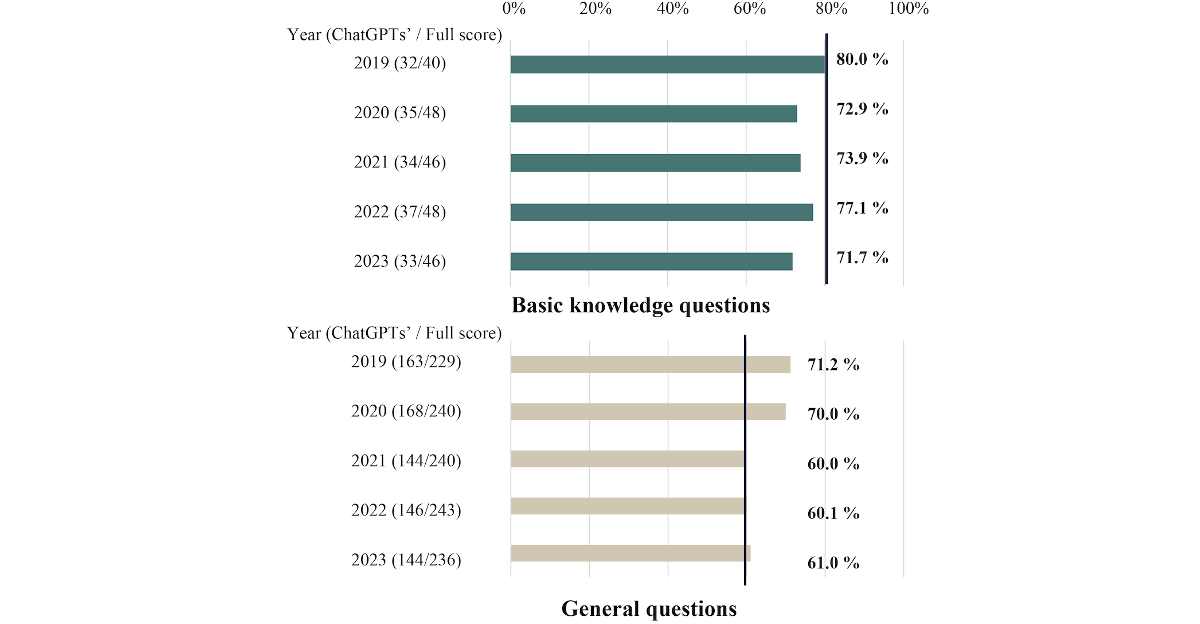
Percentages of correct scores provided by ChatGPT.

The 5-year average percentage of correct answers provided by ChatGPT was 75.1% (SD 3%) for basic knowledge questions and 64.5% (SD 5%) for general questions (Figure 1). Throughout the study period, the percentage of correct answers exceeded the passing criteria in 2019 for basic knowledge questions (passing criterion: 80%) and in all years from 2019 to 2023 for general questions (passing standard: approximately 60%). The percentage of incorrect answers per question ID tended to be higher in the morning and afternoon sessions for IDs 51-60, and in the afternoon session for IDs 91-120 (Multimedia Appendix 2). IDs 51-60 included questions in the areas of pediatric and maternal nursing and IDs 91-120 included situation-setup questions. Items with high percentages of incorrect answers included questions with complex situational settings and a combination of questions requiring the selection of 2 correct answers from a set of choices (both of which must be correct) and a situation-setup question. The percentage of incorrect answers for questions in which the options included a combination of 2 items, such as combinations of words connected by hyphens (1. A ———B, 2. C———D, 3. E———F, 4.G———H), were also high.

**Figure 2:**
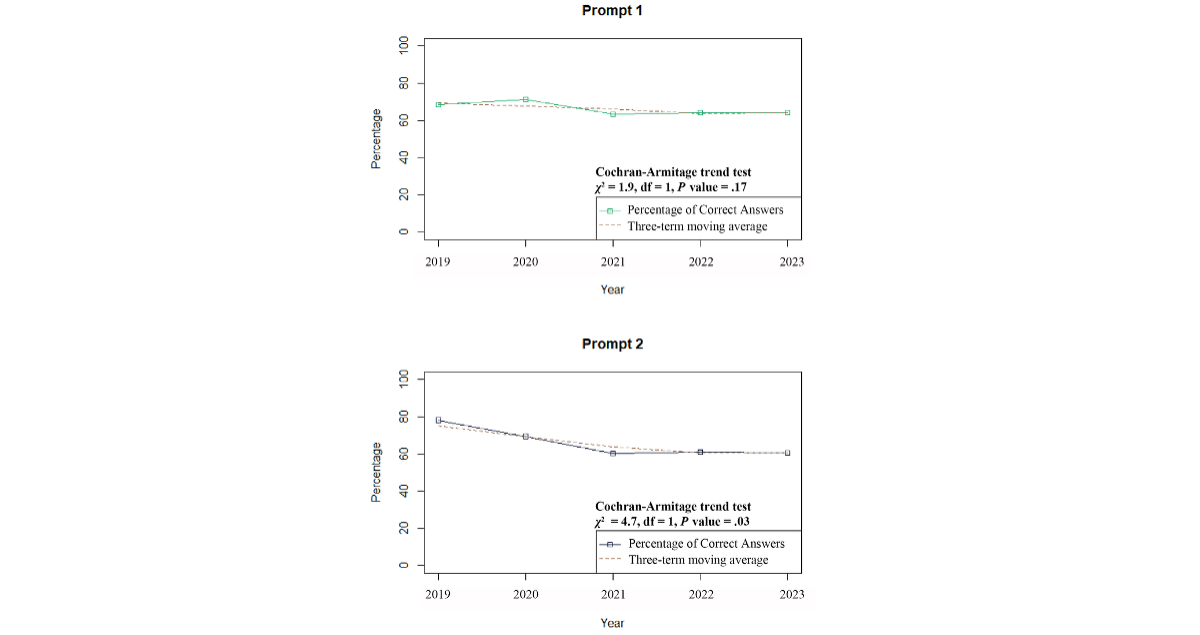
Trends in the percentage of correct answers for prompts 1 and 2.

Comparing simple-choice questions (prompt 1) and situation-setup questions (prompt 2), the average percentage of correct answers for prompt 1 was 66.3% (SD 3%) and 65.9% (SD 7%) for prompt 2. Differences in the proportions of correct answers between prompts 1 and 2 were not observed throughout the study period (Table 2). However, prompt 1 showed no significant change over time, while prompt 2 showed a gradual downward trend over time (Figure 2).

**Table 2:** Percentages of correct answers by prompt type.

			Total, n		Correct, n		Incorrect, n	Correct answers, %		*P* value (chi-square test)	
**2019**											.24
	Prompt 1	159		109		50		68.6			
	Prompt 2	55		43		12		78.2			
**2020**											.94
	Prompt 1	170		121		49		71.2			
	Prompt 2	59		41		18		69.5			
**2021**											.78
	Prompt 1	170		108		62		63.5			
	Prompt 2	58		35		23		60.3			
**2022**											.78
	Prompt 1	173		111		62		64.2			
	Prompt 2	59		36		23		61.0			
**2023**											.77
	Prompt 1	170		109		61		64.1			
	Prompt 2	56		34		22		60.7			

**Figure 3:**
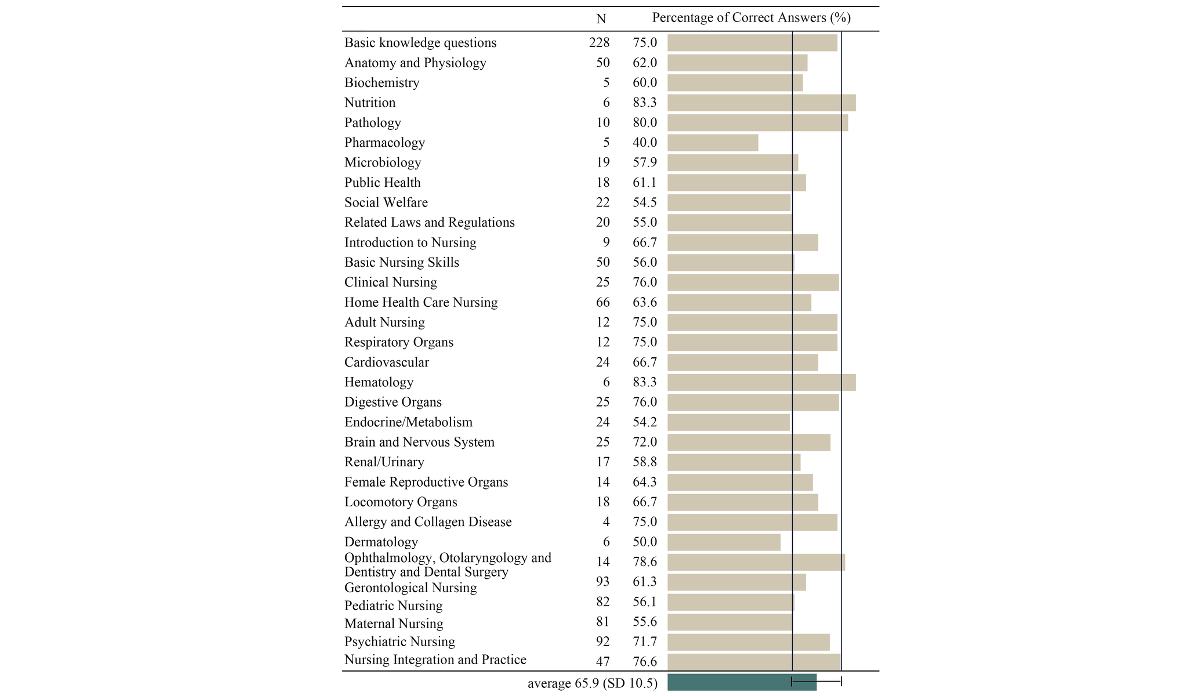
Percentages of correct answers by question area.

On comparing the percentages of correct answers for each subject area among all questions included in the analysis, the average percentage of correct answers for all areas was 65.9% (SD 10.5%; Figure 3). The subject areas with a mean value that is lower than 1 SD (55.4%) included pharmacology, social welfare, related law and regulations, endocrinology/metabolism, and dermatology. The subject areas with a mean value that is higher than 1 SD (76.4%) included nutrition, pathology, hematology, ophthalmology, otolaryngology, dentistry and dental surgery, and nursing integration and practice. ChatGPT also performed well on dialogue-related questions, with no significant difference in the percentage of correct answers to non–dialogue-related questions (*P*=.36; Multimedia Appendix 3). A dialogue question is a question in which the options are sentences enclosed in brackets; in Japanese, the brackets are the spoken words of a person.

## Discussion

### Principal Results

ChatGPT met the passing criteria for only the 2019 Japanese National Nurse Examination. Although it did not pass the 2020-2023 examinations, it scored very close to the passing criteria, with only a few more correct answers required to pass. Variations in the percentages of correct answers over the 5-year period were small, the probability of obtaining a high score by chance was low, and the performance of ChatGPT was stable. Therefore, although not significantly different, the possible reasons the percentage of correct responses tended to decrease with each passing year from 2019 to 2023 include the following: (1) lack of up-to-date data (ChatGPT only studied records until 2021) and (2) increased question complexity. Although GPT-3.5 learned data only up to 2021, it is crucial to highlight that ChatGPT is able to answer first-time questions; in other words, it is not simply filling in holes using existing internet sources, as there was no sharp decrease in scores in the 2022 and 2023 examinations. The fact that the situation-setup questions were also answered correctly without significant difference indicates that ChatGPT seemed to do well on the questions dealing with the human mind, such as those involving conversations with patients. Meanwhile, the possibility of losing track of relevant issues in complex situational settings and of having limitations such as difficulty recognizing certain expressions, including the frequent use of hyphens and other expressions, were also shown. If the current version of ChatGPT were used in nursing practice, it could be difficult to assess patients whose situations are complex, such as those requiring treatment for multiple diseases or those with socioeconomic problems. However, this is likely to depend on the amount of information that ChatGPT can store in its short-term memory, which would be resolved in the future models.

### Strengths and Limitations

This study used all questions from the 2019-2023 National Nursing Licensing Examinations in Japan, and the results were highly reliable for the performance assessment of the ChatGPT’s answers with low variability. However, this study has some limitations. First, questions with figures and tables were excluded. Although GPT-3.5, which was used to measure the performance in this study, was unable to judge figures and tables, Wang et al [12] reported that combining ChatGPT and image judgment AI could interpret radiographs, and it is highly likely that these questions will be supported in future ChatGPT updates. Second, this study did not involve advanced prompt engineering or explanatory assistance for questions or answer options. More detailed and complex prompt engineering—such as providing a question and several sample answers and then having the candidate answer them, rephrasing a question into a sentence when it uses too many hyphens or other symbols, or allowing additional exchanges rather than 1 answer per question—could have resulted in a score above the passing standard. We originally planned to validate ChatGPT’s performance in line with the actual question format, and it is important to determine whether a simple question can be answered correctly by ChatGPT. Third, it should be noted that ChatGPT is like an advanced and sophisticated autocomplete system and may not inherently understand the meaning or content of the questions entered. The degree to which ChatGPT’s expressions and responses deviate from those of humans is a subject of debate; however, ChatGPT sometimes provides completely false responses without prior warning. Therefore, it may be important to prompt ChatGPT not to answer if ChatGPT is not confident in its answer or to conduct multiple dialogues to clarify the ChatGPT's decision-making process. Finally, some of ChatGPT’s answers were misaligned between the number of choices and the content of choices, and the number of digits in computational questions was not adjusted properly. We have counted the number of questions that were misaligned between the number of choices and the content of the choices, and the number of questions included were 5 in 2023, 6 in 2022,12 in 2021, 13 in 2020, and 6 in 2019. One computational question did not adjust its digits properly in 2019 and 2020; however, this was not the case in the other years. Although there were slightly more in 2020 and 2021, there were no significant differences among other years, so the impact on the overall results is expected to be limited.

In principle, judgments were made based on the content of choices, and computational questions were judged as being correct if the formula and the results of the calculation were correct.

### Comparison With Prior Work

In general, AI using a large language model is known to perform better in English than in other languages [13], although as with the United States Medical Licensing Examination [5-7], a high percentage of correct responses for the Japanese National Nurse Examinations was observed. The National Nurse Examinations include emotion-based questions, such as those involving talking to patients, which could have been appropriately handled by ChatGPT, as it reportedly has been acquiring a human-like psychological maturity [14]. A previous study pointed out that access to medical databases was limited among the training data for ChatGPT [8], and statistical data related to health, medical care, and welfare in Japan may not have been acquired because they are provided on interactive websites such as e-Stat [15] or in PDF format, thus potentially having influenced the accuracy rate of the ChatGPT’s responses.

In the future, if additional data in the areas of poor performance are acquired and tuned so that questions and options can be understood appropriately without prompt engineering or supplementary human explanation, it is highly likely that the passing criteria will be exceeded in a stable manner. More advanced tools, such as GPT-4 or Bard (developed by Google), superseding the capabilities of ChatGPT, continue to be released and are expected to be used in many clinical situations such as diagnosis, explanation of treatments and drugs, and communication with patients. However, further research will be needed on ethical issues such as the division of roles between human nurses and AI, decision-making responsibilities, and the risks for patients when applied in clinical practice.

### Conclusions

ChatGPT passed or performed very close to the passing level on the Japanese National Nurse Examinations. With additional learning, prompt engineering, and tuning of ChatGPT, it will likely exceed the passing criteria. ChatGPT has the potential to assist nurses with decisions based on data regarding the patient’s physical condition, and to provide support for psychological issues.

## Appendix

Multimedia Appendix 1 Data Sources for the 2019–2023 Japanese National Nurse Examination.

Multimedia Appendix 2 Heatmap of correct and incorrect answers by question ID.

Multimedia Appendix 3 Comparison of percentage of correct answers between dialogue and non-dialogue questions.

## Abbreviations

AI: artificial intelligence
MHLW: Ministry of Health, Labor and Welfare
NLP: natural language processing
