# Supplementary material for: Performance of the Large Language Model ChatGPT on the National Nurse Examinations in Japan: Evaluation Study
Source: JMIR Nurs. 2023 Jun 27;6:e47305. doi: 10.2196/47305 (PMC10337249; doi:10.2196/47305)
Supplement: Multimedia Appendix 1 [file nursing_v6i1e47305_app1.docx]

Appendix 1. Data Sources for the 2019–2023 Japanese National Nurse Examination

| Questions and Answers | | |
| --- | --- | --- |
|  | 2019 | https://www.mhlw.go.jp/seisakunitsuite/bunya/kenkou_iryou/ iryou/topics/tp180511-03_04_05.html |
|  | 2020 | https://www.mhlw.go.jp/seisakunitsuite/bunya/kenkou_iryou/ iryou/topics/tp190415-03_04_05.html |
|  | 2021 | https://www.mhlw.go.jp/seisakunitsuite/bunya/kenkou_iryou/ iryou/topics/tp200414-03_04_05.html |
|  | 2022 | https://www.mhlw.go.jp/seisakunitsuite/bunya/kenkou_iryou/ iryou/topics/tp210416-03_04_05.html |
|  | 2023 | https://www.mhlw.go.jp/seisakunitsuite/bunya/kenkou_iryou/ iryou/topics/tp220421-03_04_05.html |
| Passing Criteria and Inappropriate Questions | | |
|  | 2019 | https://www.mhlw.go.jp/general/sikaku/successlist/2018/ siken03_04_05/about.html |
|  | 2020 | https://www.mhlw.go.jp/general/sikaku/successlist/2019/ siken03_04_05/about.html |
|  | 2021 | https://www.mhlw.go.jp/general/sikaku/successlist/2020/ siken03_04_05/about.html |
|  | 2022 | https://www.mhlw.go.jp/general/sikaku/successlist/2021/ siken03_04_05/about.html |
|  | 2023 | https://www.mhlw.go.jp/general/sikaku/successlist/2022/ siken03_04_05/about.html |
