# Supplementary material for: Performance of the Large Language Model ChatGPT on the National Nurse Examinations in Japan: Evaluation Study
Source: JMIR Nurs. 2023 Jun 27;6:e47305. doi: 10.2196/47305 (PMC10337249; doi:10.2196/47305)
Supplement: Multimedia Appendix 2 [file nursing_v6i1e47305_app2.docx]

Appendix 2. Heatmap of correct and incorrect answers by question ID

Black filled cells are incorrect answers; gray filled cells are questions excluded from the analysis (inappropriate questions or questions with figure and table); Blue boxes are two-choice questions; red boxes are questions with options hyphenating two words.
