# Supplementary material for: Performance of the Large Language Model ChatGPT on the National Nurse Examinations in Japan: Evaluation Study
Source: JMIR Nurs. 2023 Jun 27;6:e47305. doi: 10.2196/47305 (PMC10337249; doi:10.2196/47305)
Supplement: Multimedia Appendix 3 [file nursing_v6i1e47305_app3.docx]

Appendix 3. Comparison of percentage of correct answers between dialogue and non-dialogue questions

|  | Correct | | Incorrect | | Chi-square value | p-value^a^ |
| --- | --- | --- | --- | --- | --- | --- |
| Dialogues^b^ | 57 | (61.3) | 36 | (38.7) | 0.85 | 0.36 |
| Non–Dialogues^b^ | 690 | (66.6) | 346 | (33.4) |  |  |
| Total | 747 | (66.2) | 382 | (33.8) |  |  |

^a^Chi-square test; ^b^dialogue only includes questions with options that have brackets indicating the nurse's lines.
